# Sarcolab pilot study into skeletal muscle’s adaptation to long-term spaceflight

**DOI:** 10.1038/s41526-018-0052-1

**Published:** 2018-09-17

**Authors:** Jörn Rittweger, Kirsten Albracht, Martin Flück, Severin Ruoss, Lorenza Brocca, Emanuela Longa, Manuela Moriggi, Olivier Seynnes, Irene Di Giulio, Leonardo Tenori, Alessia Vignoli, Miriam Capri, Cecilia Gelfi, Claudio Luchinat, Claudio Franceschi, Roberto Bottinelli, Paolo Cerretelli, Marco Narici

**Affiliations:** 1 Institute of Aerospace Medicine, German Aerospace Center (DLR), Cologne, Germany; 2 Department of Pediatrics and Adolescent Medicine, University of Cologne, Cologne, Germany; 3 Faculty of Medical Engineering and Technomathematics, FH Aachen University of Applied Science Aachen, Aachen, Germany; 4 Institute of Biomechanics and Orthopaedics, German Sport University, Cologne, Germany; 5 Department of Orthopaedics, University of Zürich, Zürich, Switzerland; 6 Department of Molecular Medicine, University of Pavia, Pavia, Italy; 7 CNR-IBFM, Segrate, MI Italy; 8 Department of Physical Performance, Norwegian School of Sport Sciences, Oslo, Norway; 9 Centre for Human and Applied Physiological Sciences, King’s College London, London, UK; 10 Department of Experimental and Clinical Medicine, University of Florence, Florence, Italy; 11 CERM Centro di Ricerca di Risonanze Magnetiche, Florence, Italy; 12 Department of Experimental, Diagnostic and Specialty Medicine, University of Bologna, Bologna, Italy; 13 Department of Biomedical Sciences for Health, University of Milan, Milano, Italy; 14 Fondazione Salvatore Maugeri (IRCSS), Scientific Institute of Pavia, Pavia, Italy; 15 Department of Biomedical Sciences, University of Padova, Padova, Italy

## Abstract

Spaceflight causes muscle wasting. The Sarcolab pilot study investigated two astronauts with regards to plantar flexor muscle size, architecture, and function, and to the underlying molecular adaptations in order to further the understanding of muscular responses to spaceflight and exercise countermeasures. Two crew members (A and B) spent 6 months in space. Crew member A trained less vigorously than B. Postflight, A showed substantial decrements in plantar flexor volume, muscle architecture, in strength and in fiber contractility, which was strongly mitigated in B. The difference between these crew members closely reflected FAK-Y397 abundance, a molecular marker of muscle’s loading history. Moreover, crew member A showed downregulation of contractile proteins and enzymes of anaerobic metabolism, as well as of systemic markers of energy and protein metabolism. However, both crew members exhibited decrements in muscular aerobic metabolism and phosphate high energy transfer. We conclude that countermeasures can be effective, particularly when resistive forces are of sufficient magnitude. However, to fully prevent space-related muscular deterioration, intersubject variability must be understood, and intensive exercise countermeasures programs seem mandatory. Finally, proteomic and metabolomic analyses suggest that exercise benefits in space may go beyond mere maintenance of muscle mass, but rather extend to the level of organismic metabolism.

## Muscles: Onboard exercise limits muscle loss in space

Physical activity with resistive forces helps preserve muscle volume, architecture and strength in space. A team led by Jörn Rittweger from the German Aerospace Center in Cologne studied two crew members who spent six months on board the International Space Station. During the Sarcolab pilot study, one of these astronauts performed less exercise than the other. After returning to Earth, the one who trained less showed more substantial deterioration of the plantar flexor muscle in the foot—a difference detectable also at the molecular level, with lower levels of proteins involved in anaerobic and aerobic muscle metabolism. The findings highlight the need to vigorously exercise in space to limit muscle weakness. Doing so does not seem to fully prevent space-related problems, though, as evidenced by signs of muscle wasting even in the astronaut who trained regularly.

## Introduction

Physical deconditioning is known to occur during spaceflight since Skylab and Mir missions. Lower limb muscles, in particular, undergo rapid wasting and loss of function.^1,2^ Most affected is the triceps surae muscle, where 20% fiber atrophy occurs after 6 months of spaceflight.^3,4^ In ground-based models (e.g. bed rest), muscle atrophy is associated with decreases in fascicle pennation angle and length.^5,6^ Alterations of muscle architecture are expected to affect the mechanical output, thereby contributing to muscle weakness.^7^ Moreover, muscle unloading also leads to reductions in the fibers’ specific force and power, and in myosin heavy chain (MCH) concentration.^8^ All these factors can independently alter the mechanical capabilities of muscles.

Muscle atrophy results from imbalance between protein synthesis and degradation. This imbalance can be caused by enhanced muscle protein breakdown (MPB), controlled by catabolic pathways (ubiquitin proteasome and autophagy), and also by inhibited muscle protein synthesis (MPS), controlled by the Akt/mTOR/p70S6K pathway.^9^ To date, the relative contribution of MPB and MPS is still unclear.^10^ The determination of the actual rates of protein synthesis and degradation in humans is challenging. The relative activation of intracellular pathways involved varies across species and disuse conditions. Moreover, it is unclear whether a metabolic program plays a relevant role in causing disuse atrophy in humans. Recently, it has been suggested that mitochondrial dysfunction is a major trigger of MPB and MPS imbalance.^11,12^ Solving these open issues is important for spaceflight.

Countermeasure exercises are nowadays mandatory on board the International Space Station (ISS). With regards to muscle, they involve exercises with the advanced resistive exercise device (aRED) and a treadmill (T2). Effects of strength training upon skeletal muscle on Earth have been studied extensively, but relatively little is known about the molecular events in disuse or in spaceflight.^13^ It is therefore an open question how far training in space is helpful to maintain the lower limb musculature.

Costameric proteins can serve as a molecular proxy of the muscle’s loading history.^14–20^ They anchor the sarcomeres to extracellular matrix receptors.^21–23^ Among these, the integrin-linked focal adhesion kinase (FAK) is a mechanically regulated costamere component that controls the turnover of focal adhesion in a fiber type-specific manner together with FAK-related non-kinase FRNK, FAK’s natural inhibitor.^18,21,24^ Herein, the content of post-translation modification of tyrosine 397 (Y397) is a critical event, which is affecting the activity of FAK.^25–27^ Importantly, the FAK pathway likely controls protein synthesis through 70S6K, a component of the Akt/mTOR pathway.

Thus, the Sarcolab study has been designed to: (i) disentangle the various constituents of muscle weakness associated with spaceflight; (ii) elucidate the muscular adaptations to long-term spaceflight; (iii) address the affected molecular and metabolic pathways in skeletal muscle and in the blood in astronauts during long missions on ISS. The main hypothesis of the Sarcolab study is that alterations in the physiological cross-sectional area, fiber length, as well as in single fiber mechanics collectively contribute to the space-related muscle weakness. Moreover, the study aims to screen for muscle proteomic adaptations to spaceflight. Finally, intracellular signaling pathways, namely those controlling muscle mass (ubiquitin proteasome pathway, autophagy, FAK) and metabolism (PGC-1alpha, SREBP-1) are studied, and blood metabolomics screens for systemic consequences-related muscular alterations. The present paper reports results from two astronauts who took part in the Sarcolab pilot study, before the experiment had been enlarged into the currently performed three-agency study (Sarcolab3), supported by ESA, NASA, and Roscosmos.

## Results

### Onboard exercise training

**Fig. 1:**
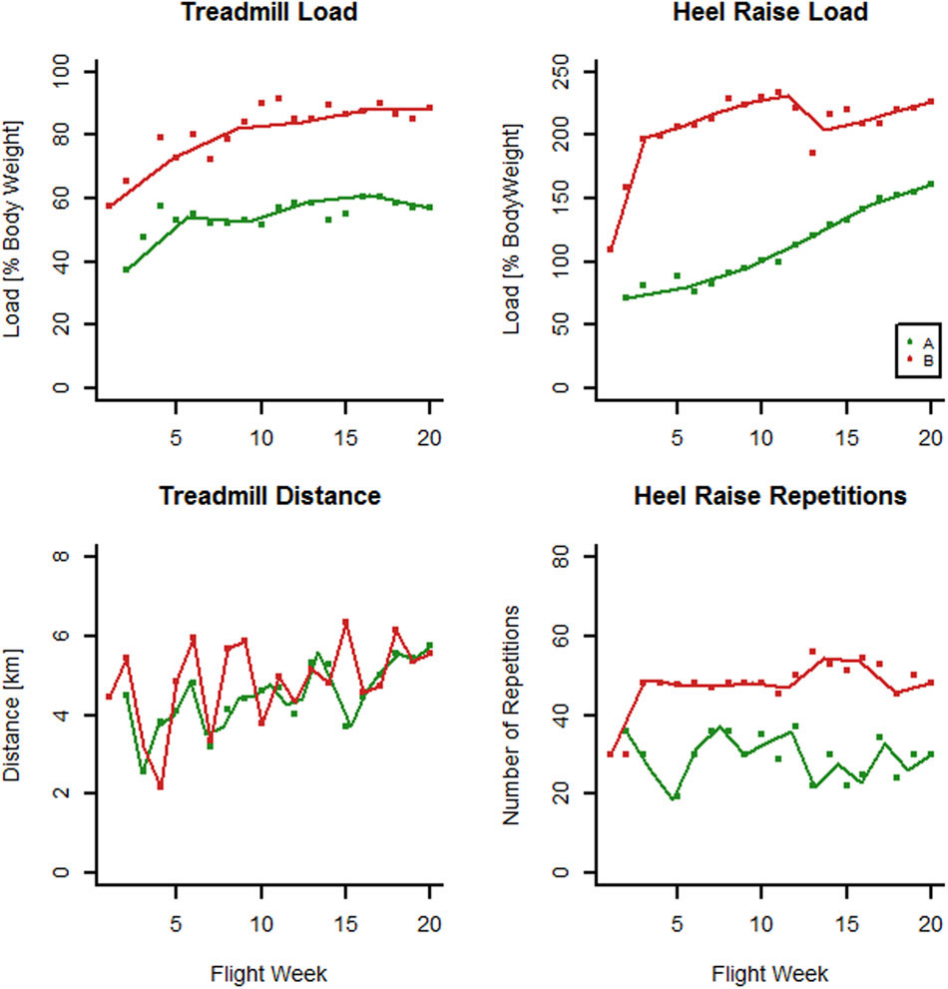
Onboard exercise. Survey of the load and distance per treadmill session, and the load and number of heel raise exercise with aRED for crew members A and B during their sojourn on the ISS. Data are means for each week on board ISS, interpolated by a spline function

Crew member A performed fewer treadmill sessions than B (90 vs. 114), ran with lower pull-down force (median of 55.9 vs. 85.6% of body weight), ran at slower speed (median 11.3 vs. 12.9 km/h), and covered a shorter distance than B per running session (median 4.7 vs. 5.8 km, Fig. 1). Crew member A also trained less with aRED, performing fewer heel raise sessions (54 vs. 98) with fewer repetitions (median 30 vs. 48) and at lower resistive force (median 122 vs. 221% of body weight). Thus, A was generally training less vigorously than B, in particular with regards to exercise elements related to loading force.

### Muscle function

**Fig. 2:**
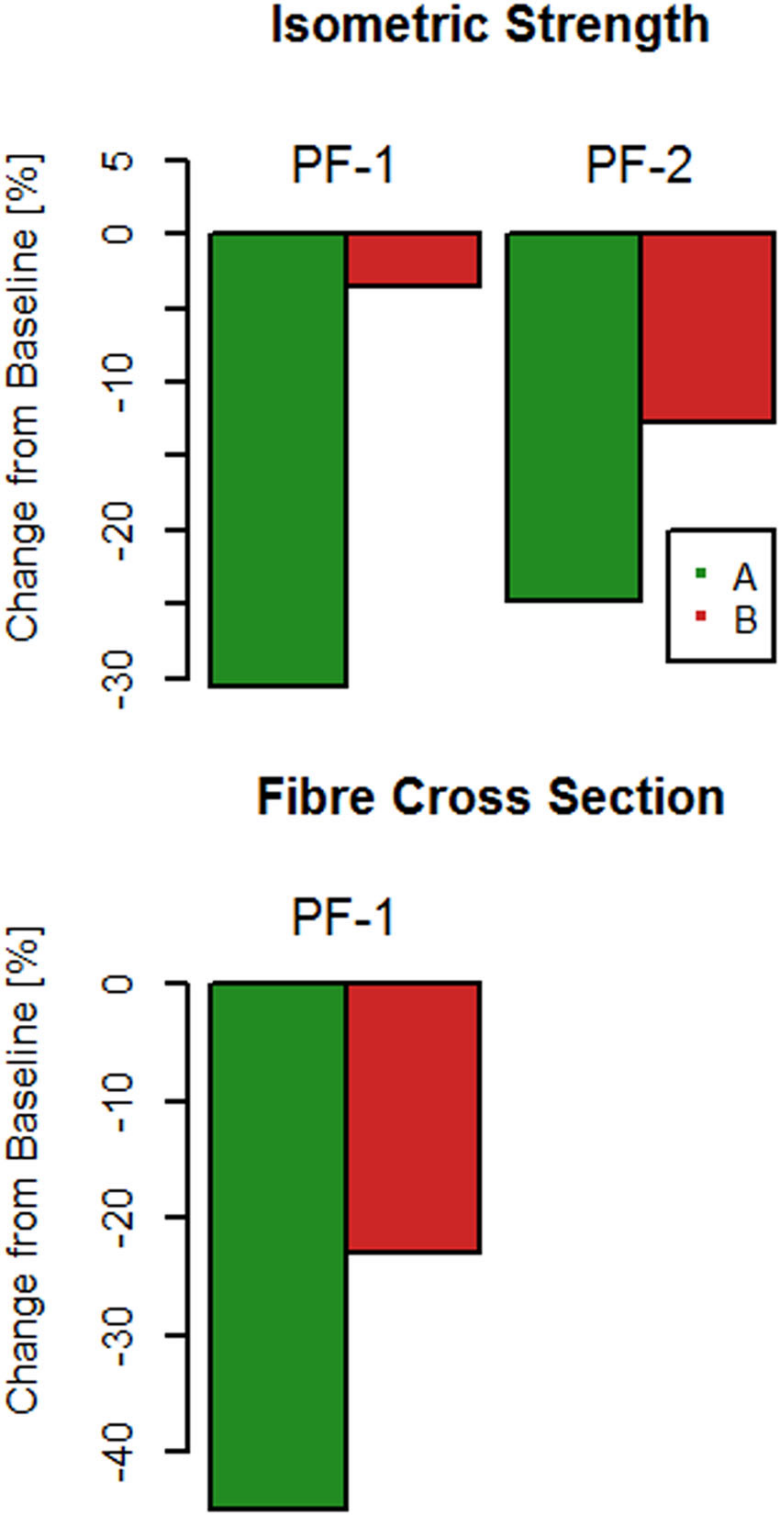
Effects of spaceflight upon muscle function. Data are given in percent changes from baseline, for crew members A and B

During postflight session 1 (PF-1, performed 0−4 days after return to Earth), plantar flexor muscle strength was reduced by 30.6% in crew member A, whereas B depicted no change from baseline (Fig. 2).

### Muscle size and architecture

Medial gastrocnemius (GM) muscle volume, pennation angle (PA), and fascicle length (Lf) at PF-1 were all substantially reduced in crew member A, but much less in B (Table 1). Physiological cross-sectional area (pCSA = Vol/Lf) was affected to comparable extent in both crew members at PF-1 (Table 1). At PF-2 (performed at R + 15), pCSA of the GM muscle had recovered in B, but not in A.

**Table 1:** Changes in muscle size and architecture

	Crew Member	PF-1	PF-2
**MEDIAL GASTROCNEMIUS MUSCLE**			
Volume	A	−18.1%	−10.7%
	B	−7.6%	−4.4%
Pennation angle	A	−23.2%	−13.1%
	B	3.7%	3.3%
Fascicle length	A	−7.6%	−0.6%
	B	−1.3%	−0.6%
Physiological CSA	A	−11.4%	−10.2%
	B	−6.4%	−3.8%
**SOLEUS MUSCLE**			
Volume	A	−21.1%	−16.6%
	B	−9.8%	−8.2%
Pennation Angle	A		
	B	−2.9%	6.2%
Fascicle length	A		
	B	0.6%	1.7%
Physiological CSA	A		
	B	−10.3%	−9.7%

Data represent percent changes from baseline for testing sessions postflight 1 (PF-1, at 0−4 days after landing) and postflight 2 (PF-2, at 15 days after landing). Soleus muscle architecture could not be analyzed in crew member A, only in B

### Fiber type, fiber size and fiber mechanics

At baseline, soleus (SOL) muscle was almost exclusively composed of the slow isoform of myosin heavy chain (MHC-1) in both crew members. No shift in MHC relative content was observed postflight. Cross-sectional area of single muscle fibers (CSA_SF) at PF-1 declined in crew member A by −45%, but only by −23% in B (Fig. 2).

Functional single fiber analysis could be performed pre- and postflight in crew member A only. All fibers analyzed were type 1 fibers. Specific isometric force (Po/CSA) was lower postflight than preflight (−27%). Unloaded shortening velocity (Vo) as well as actin sliding velocity (Vf) were both comparable pre- vs. postflight, suggesting no alteration in the actomyosin kinetics and no alteration of myosin at molecular level.

### Costameric protein expression

All costameric proteins studied were detectable in SOL at baseline (Figures S1 to S3). Gamma-vinculin was more abundant than meta-vinculin (Figure S1). At PF-1, protein concentrations of FAK (normalized to actin) was reduced in crew members A and B by −60 and −44%, respectively, and FRNK by −60 and −67% (Table 2, Figure S2). By contrast, FAK and FRNK concentrations were both increased above baseline levels at PF-2 (Table 2, Figure S2). Importantly, FAK-pY397 concentration was decreased in crew member A at PF-1, but increased in B (Figure S2, Table 2). These findings suggest that musculoskeletal forces in space may have been comparable to those on Earth in crew member B, but substantially reduced in A.

**Table 2:** Summary of expressional alterations in costamere proteins

Parameter	PF-1		PF-2	
	Crew member A	Crew member B	Crew member A	Crew member B
FAK-pY397	−92%	11%	−54%	28%
FAK	−60%	−44%	112%	88%
FRNK	−60%	−67%	124%	26%
Gamma-vinculin	5%	−17%	4%	23%
Meta-vinculin	786%	−26%	−24%	116%
Tenascin-C	2781%	−14%	−40%	64%

Data are given as percent changes from baseline

Concentration of the meta-vinculin protein isoform and tenascin-C was increased at PF-1 in crew member A by 786 and 2781%, respectively, but both were slightly reduced in crew member B (Table 2, Figures S1 and S3). At PF-2, these proteins resumed to near baseline levels in crew member A, but were increased in crew member B (Figure S1). The concentration of gamma-vinculin was only marginally affected by spaceflight (Table 2).

Baseline meta-vinculin:gamma-vinculin ratio was comparable in crew members A and B. Crew member A depicted eightfold and fivefold increases at PF-1 and PF-2, respectively, but no changes were observed in B (Table S1). Baseline FRNK:FAK ratio was greater in crew member A than B (2.01 vs. 0.58). It remained unchanged in crew member A after spaceflight, but decreased moderately in B.

### Skeletal muscle proteomic analysis

From 1100 spots detected in the two-dimensional difference in gel electrophoresis 2D-DIGE, 900 were included in the base set for statistical analysis. The principal component analysis (PCA) of the muscle tissue from both crew members yielded two components (PCA1 and PCA2) that explain 59.2 and 25.9% of the global variation, respectively (Figure S4). Concordant changes were observed for PCA2 from baseline to PF-1 in both crew members, but changes were discordant from baseline to PF-2, indicating closer similarity between PF-2 and baseline in crew member B.

**Fig. 3:**
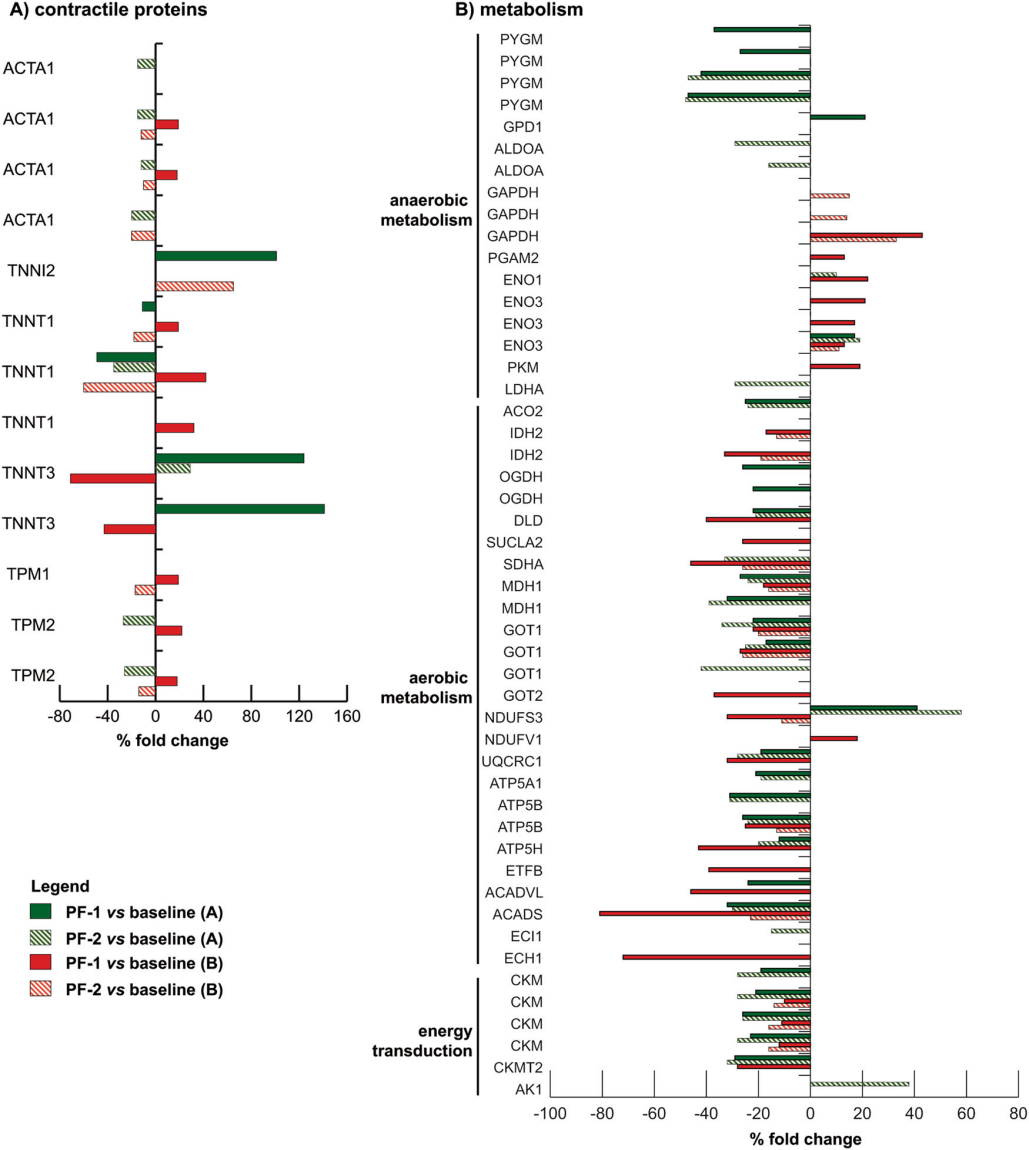
Proteomic analysis in human skeletal muscle. Histograms of differential protein expression in soleus muscle between baseline vs. PF-1 (colored bars) and baseline vs. PF-2 (striped bars) in crew member A (green bars) and B (red bars), as detected by 2D DIGE analysis. Proteins significantly altered (paired one-way ANOVA and Tukey, *α* = 0.01) are indicated by their gene name and expressed as a percent of spot volume variation. **a** Contractile proteins; **b** Metabolism

Proteomic analyses followed by paired one-way ANOVA and Tukey tests (*α* = 0.01) indicated significant differences between baseline and PF-1 in 32 and 39 spots in crew members A and B, respectively. When comparing baseline to PF-2, 37 spots changed in crew member A and 24 spots in crew member B (Fig. 3 and Table S2).

Concerning *contractile proteins*, PF-1 samples revealed alterations in troponin I and troponin T in both crew members, but only crew member B revealed some moderate changes in actin and tropomyosin (Table S2). More specifically, crew member A demonstrated distinct increases in troponin I fast (TNNI2) and in two proteoforms of troponin T fast (TNNT3), and decreases in two proteoforms of troponin T slow (TNNT1). Notably, where PF-1 changes in the troponins were observed in B, they were in the opposite direction in crew member A. At PF-2, some moderate decreases in actin proteoforms (ACTA1) were observed in both crew members.

Proteins involved in *anaerobic metabolism* were also affected by spaceflight. Crew member A depicted decreases in four different proteoforms of glycogen phosphorylase (PYGM) at PF-1, as well as increases in glycerol-3-phosphate dehydrogenase (GPD1) and in one beta-enolase proteoform (ENO3). By contrast, B depicted increases in glyceraldehyde-3-phosphatase dehydrogenase (GAPDH), phosphoglycerate mutase 2 (PGAM2), alpha-enolase (ENO1), three proteoforms of ENO3 and pyruvate kinase (PKM). At PF-2, two of the PYGM proteoforms were recovered in crew member A, but decreases in two proteoforms of fructose-bisphosphate aldolase A (ALDOA) and l-lactate dehydrogenase A chain (LDHA) occurred at that time. Thus, dysregulation of glycogen metabolism and accumulation of specific enolase proteoforms appeared postflight in crew member A, whereas crew member B adapted metabolically to spaceflight by increasing anaerobic metabolism.

Concerning *aerobic metabolism*, enzymes involved in malate shuttle, in oxidative phosphorylation and in lipid metabolism were downregulated in both crew members at PF-1 and at PF-2. In crew member A, this encompassed virtually all enzymes, except that NADH dehydrogenase iron-sulfur protein 3 (NDUFS3) was upregulated. Crew member B depicted a very similar response as A, except that NDUFS3 was down- rather than upregulated (Table S1). Thus, aerobic metabolism seemed comparably compromised in both crew members.

Enzymes involved in high *energy phosphate transfer* likewise depicted a downregulation in both crew members postflight. This was more pronounced in crew member A than B. The enzymes affected involved four proteoforms of creatine kinase M-type (CKM) and creatine kinase S-type (CKMT2). Overall, dysregulation of high energy phosphate production could be a consequence of the severe impairment of aerobic metabolism, or vice versa.

### Intracellular signaling pathways controlling muscle mass and metabolism

The two major catabolic systems were studied on mRNA level by assessing expression of MuRF-1 and atrogin-1 (markers of the ubiquitin proteasome activity) and of p62 and Beclin-1 (markers of autophagy). Whereas atrogin-1 expression was upregulated postflight in both crew members, MuRF-1 expression was highly upregulated in crew member A only. Beclin-1 was upregulated in both crew members postflight, whereas p62 was upregulated in crew member A only.

At PF-2, expression of all of these markers was lower than at PF-1. In crew member A, recovery towards normal activation was somewhat less complete, especially for MuRF-1. The results suggest a higher activation of both catabolic systems and a slower recovery towards normal values in crew member A.

PGC-1alpha is a master controller of mitochondrial biosynthesis and oxidative metabolism. It was surprisingly upregulated in both crew members at PF-1, and even at PF-2. SREBP-1 is a transcription factor controlling lipid synthesis. It was upregulated in crew member B only.

NRF2 is a transcription factor involved in the response to injury and inflammation, and thus also in sensing the level of intracellular ROS and stimulates synthesis of antioxidant defense systems. Its increase occurred in crew member B only, where it was still observed at PF-2.

### Plasma metabolomics

**Fig. 4:**
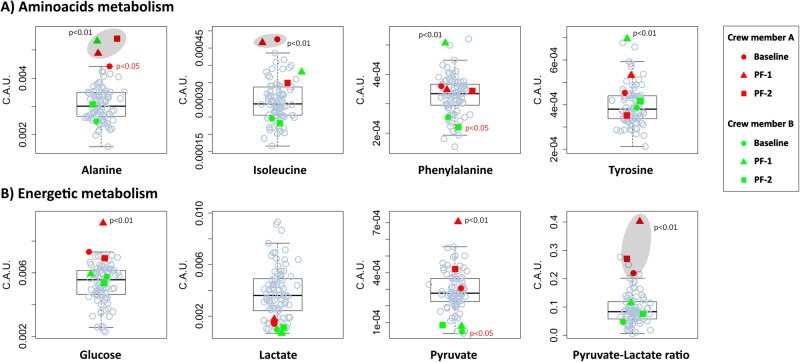
Metabolomic analysis. Panels **a**, **b** show amino acid and energetic metabolites, respectively, in box plot analysis and in arbitrary units concentrations (C.A.U.) of the most significant metabolites. Astronauts A and B are represented by red and green colors, respectively. Circle, square, and triangle represent baseline, PF-1 and PF-2, respectively. Gray circles represent the control cohort (79 volunteers). Significant (*P* < 0.01) and barely significant (*P* < 0.05) *P* values are also reported

Twenty metabolites were assigned and quantified in the spectra. Levels of significant metabolites (plasma amino acids, glucose, lactate, pyruvate, including the pyruvate/lactate ratio) are reported along with their *P* values in Fig. 4, compared with a cohort of 79 control subjects. All baseline metabolite levels were comparable with control data, except that isoleucine was elevated and alanine was at the upper control margin at PF-1 in crew member A. Serum alanine was increased in this crew member postflight (in both PF-1 and PF-2 *P* < 0.01), but conversely from isoleucine was not normalized at PF-2. Moreover, crew member A depicted elevated serum levels of glucose and pyruvate, as well as an elevated pyruvate-lactate ratio on day PF-1 (all *P* < 0.01).

In crew member B, serum levels were generally within the control margins. The only exceptions were alanine, phenylalanine, and tyrosine, which all increased at PF-1 (*P* < 0.01), but not at PF-2.

## Discussion

The present study provides a multifaceted account of strongly diverging responses to spaceflight in two astronauts. While crew member A exhibited the expected neuromuscular responses, namely decrements in plantar flexor muscle volume, altered architecture, reductions in contractile protein composition, downgraded fiber contractility, and thus overall muscle strength, crew member B was much less affected after long-term spaceflight. Notably, the loading levels achieved by crew member B are rarely seen in other crew members on ISS. It is therefore tempting to ascribe the salient differences between A and B to the different muscular exercises, in particular with regards to resistive forces. One could therefore ascribe the divergent muscle proteomic responses (contractile proteins and aerobic metabolism affected in crew member A, but almost not in B), as well as differences in the response of the catabolic pathways (greater activation of ubiquitin proteasome system and of autophagy in crew member A), and in systemic metabolic profiles (greater effects upon energy and protein metabolism in A than in B) to the different countermeasure regimens. These findings therefore underline the importance of the onboard exercise. However, the 23% decrement in muscle fiber CSA coupled to the activation of catabolic pathways that was still occurring in crew member B, and the fact that aerobic metabolism and high phosphate energy transfer were also compromised in crew member B may suggest that complete preservation of the musculature in space is not yet achievable.

It is also intriguing that the response of the load-dependent parameters, pY397-FAK and meta-vinculin concentration, but not FAK or FRNK, reflect the diverging responses in muscle mass and strength.^17,18^ The observed downregulation of FAK and FRNK protein levels at PF-1 replicate earlier findings in antigravity muscles of rats and humans, implicating a requirement of gravitational loading to maintain the gene products of the FAK gene (i.e. PTK2).^16–18^ The present observations confirm that muscle response to unloading involves a net reduction in the capacity for regulation of costamere turnover via Y397 phosphorylated FAK.^15^ Consistent with this notion, the concentration of both FAK and FRNK increased 2 weeks after return to Earth, indicating reestablishment of adhesion sites in the sarcolemma with resumption of load-bearing muscle activity. Alterations in the proxy of costamere remodeling, FAK-pY397, were inversely related to alterations in the concentration of the costameric protein meta-vinculin and tenascin-C at PF-1 (Table 2; Figures S1−S3). At this time point, and in absolute terms at postflight session 2, expression of meta-vinculin and tenascin-C postflight was considerably less affected in crew member B, while FAK-pY397 concentration was selectively increased in this crew member. This is consistent with the reported upregulation of FAK-pY397 concentration by muscle loading and the greater extent of muscle loading of crew member B than A during the onboard exercise (Fig. 1).^28,29^ Collectively, the study of costamere-associated proteins suggests protection of the soleus muscle in crew member B inflight, but muscle damage due to unaccustomed mechanical loading after return to Earth.

Importantly, the adaptations in the FAK pathway, modulating the anabolic Akt/mTOR/p70S6K pathway, can link the unloading and muscle atrophy to the imbalance between protein synthesis and degradation.^30,31^

Interestingly, muscular aerobic metabolism was compromised in both crew members, notwithstanding the extensive treadmill exercise performed by crew member B and the enhanced expression of PGC-1alpha. PCG-1alpha enhances mitochondrial biosynthesis and oxidative metabolism and is a key regulator in the metabolic response to endurance training.^32,33^ In disuse, PGC-1alpha expression is reduced in soleus muscle of mice and in vastus lateralis muscle of humans.^12,34^ Reduced expression of PGC-1alpha has been considered a relevant cause of muscle atrophy, suggesting a “metabolic” program of muscle atrophy.^11,12,34^ The higher expression of PGC-1alpha in this study is surprising. Possibly, inflight countermeasures have prevented PGC-1alpha downregulation. Alternatively, PGC-1alpha upregulation could be an unsuccessful attempt to counteract the decrease in energy levels, namely an increase in AMP and a decrease in ATP due to impaired energy metabolism.

Expression of SREBP-1, a master regulator of lipid metabolism, was increased in the soleus muscle in both crew members after spaceflight. This is consistent with the observation that weightlessness induces intramuscular lipid accumulation.^35^ Furthermore, it is interesting that NRF2, a transcription factor sensitive to intracellular ROS and simultaneously a stimulator of antioxidant defense systems, was elevated in crew member B, but not in crew member A. Redox imbalance had been observed in human bed rest and in hindlimb suspended mice.^12,34,36^ However, as crew member B performed more exercise and had less muscle atrophy than A, the higher NRF2 expression in B could be due to an aerobically more intense exercise regimen in B than in A, inasmuch as oxygen demand in running increases with loading forces.^37^ Given that NRF2 and proteoforms related to mitochondrial and aerobic metabolism were divergently affected by space, one might thus even speculate about a relative overemphasis of aerobic exercise, similar to overtraining on Earth.

Both crew members diverged also in their metabolomic profiles. While postflight changes in crew member A were more pronounced for energy than for amino acid metabolism, the opposite was true for crew member B. Moreover, the increases in glucose, pyruvate, and pyruvate/lactate ratio in crew member A can be understood as the metabolic consequence of muscle wasting.^38^ Interestingly, both crew members’ metabolic profiles appeared distinct already before launch, as shown by PCA. During spaceflight, they responded in two different ways, moving toward different positions at PF-1, and finally both reverted to their initial metabolic spaces at PF-2 (Figure S5). This behavior is consistent with the observed metabolic resilience previously described in healthy controls.^39^

Collectively, these data show distinctly diverging responses in two crew members that performed countermeasure exercises at different intensities during their half-year sojourns on ISS, underlining the need of physical exercises in space. The findings also show that running as much as 500 km, with a resistive force close to the body weight, and performing 5000 heel raises at a force of more than twice the body weight was insufficient to entirely prevent muscle atrophy and weakness. Upregulation of the ubiquitin proteasome pathway and increased autophagy likely contributed to muscle wasting, more so in crew member A than B. Upregulation of PGC-1alpha was also found in both crew members, suggesting that the aerobic exercise performed onboard may have been sufficient to prevent disuse-induced decrease in PGC-1alpha, but not the impairment in metabolism and its impact on muscle integrity. The atrophy found both at whole muscle and single fiber level (mostly in crew member A) was associated with a reduction in FAK content and, in crew member A, also in phosphorylated FAK. Instead, in crew member B who exercised very intensively, FAK-pY397 levels actually increased. We have shown in previous ground-based studies that FAK plays an essential role in skeletal muscle remodeling with use, disuse and ageing and the present results confirm its role in regulating muscle mass.^31,40–42^ Adaptations of FAK pathway could, in fact, inhibit protein synthesis and contribute to muscle atrophy. Finally, proteomic assessment of muscle tissue and metabolomic blood analyses indicate that exercise may have benefits in space that go far beyond mere maintenance of muscle mass and strength.

## Methods

### Subjects and testing schedule

Two crew members of equal sex and comparable age, A and B, were tested before and after their half-year ISS mission (Table S3). The methods were performed in accordance with relevant guidelines and regulations and approved by the Human Research Multilateral Review board, and written informed consent was obtained prior to study inclusion. Inflight countermeasure exercises data were obtained via data sharing with NASA. Heel raise exercises on aRED were computed to yield the total number of repetitions, and peak and average resistive force per session was normalized to body weight. For treadmill data, distance, speed, exercise time, and the bungee force at rest were assessed.

### Plantar flexor muscle function

Muscle strength was assessed using a custom-made dynamometer.^43^ Subjects were seated upright with hip and knee joints at 90°, performing maximum voluntary isometric contractions at ankle joint angles of −20, −10, 0, 10, and 20° (0° representing the neutral position, and positive angles indicating plantarflexed positions).

### Muscle volume and architecture

Fiber length and pennation angle of the resting GM and SOL were evaluated with a MyLab25 ultrasonography scanner (Esaote Biomedica), using a 50 mm linear array probe LA523 (13-4 MHz). Scans were taken at 50% of the GM muscle length in the midsagittal plane at 90° knee flexion and −10° ankle dorsiflexion.

Muscle volume was assessed via transversal magnetic resonance images, with 3 mm slice thickness and 3 mm gaps. Before acquisition, subjects laid supine for 30 min.^44^ For analysis, muscles were segmented manually in every recorded slice.

### Muscle biopsy

Approximately 50 mg of tissue were harvested from the soleus muscle with an 11G ACECUT automatic biopsy system (TSK Laboratory, Oisterwijk, the Netherlands). A lateral approach was chosen approximately 2 cm below the distal end of the lateral gastrocnemius muscle. Before incision, the skin was razed and disinfected, and local anesthetic (Lidocain 1%) was injected. Samples were divided and aliquoted within 10 min after the biopsy.

*MHC isoform distribution* was assessed as described.^45–47^ Single muscle fiber segments were dissolved in Laemmli solution and loaded on 6% SDS-PAGE polyacrylamide gels.^48^ To assess MHC isoform composition in whole biopsy, frozen portion of biopsy was pulverized with liquid nitrogen and resuspended in Laemmli solution. About 15 µg of proteins were loaded on 6% SDS-PAGE polyacrylamide gels, and electrophoresis was run overnight at 100 V and the gels were stained with Coomassie Blue stain.^47,49^

### Single fiber mechanics

Fiber cross-sectional area (CSA), force, and maximum shortening velocity was assessed as previously described.^45,50^ Briefly, biopsy samples were divided in small bundles and stored at −20 °C for up to 3 weeks in skinning solution (150 mM K_2_HPO_4_, 5 mM KH_2_PO_4_, 5 mM magnesium acetate, 1 mM DTT, 5 mM EGTA, 3 mM Na_2_-ATP, pH 7, leupeptin hydrochloride 20 μg/ml, E64 10 μM) and glycerol 50%. On the day of experiment, segments of single fibers, approximately 1 mm long, were manually isolated under stereomicroscopic control at ×20–×40 magnification in skinning solution. Fibers were immersed for 1 h in skinning solution containing 0.1% Triton X-100 before functional analysis. Each fiber was mounted between a force transducer’s and an electromagnetic puller’s hook. Fiber width and depth were measured with an inverted microscope at ×320 magnification at three different positions along the fiber. CSA was determined assuming an elliptical shape. Isometric force (Po) and unloaded shortening velocity (Vo) were measured by the slack test technique. Activating solution had the following composition: 100 mM KCl, 20 mM imidazole, 5 mM MgCl_2_, 5 mM Na_2_-ATP, 0.5 mM EGTA, 25 mM creatine phosphate, 300 U/ml creatine kinase, pCa 8.0. Experiments were performed at 12 °C, in conditions of maximal activation (pCa 4.5) and at optimal sarcomere length (2.5 μm) for force development. At the end of the mechanical experiments, fibers were characterized on the basis of MHC isoform composition.

### Costameric protein biochemistry

Biopsies were sectioned and protein was extracted with the help of a rotorstat mixer (Kinematica, Lucerne, Switzerland) using RIPA buffer, and total protein quantified essentially as described.^18^ Homogenate corresponding to 10 μg total protein was separated on 7.5% SDS-PAGE gels, blotted onto nitrocellulose membrane and subjected to immunological protein detection as described using the following antibodies: 1:1000 dilution of polycolonal FAK antiserum Lulu, 1:500 of monoclonal Tenascin-C serum B28:13, 1:100 of monoclonal antiserum against gamma-vinculin and meta-vinculin (gift from Dr. M.A. Glukhova, Paris, France).^18,21,51^ Detection of pY397-FAK content was carried out based on immune-precipitation of soluble proteins in homogenate corresponding to 1 mg total protein essentially as described.^18^ Samples being derived from the three samples of each subject were analyzed in adjacent lanes of the same SDS-PAGE gel. Equal loading and blotting was verified via signal intensity of the actin band on the Ponceau S-stained nitrocellulose membrane; after blotting signal intensity was assessed from background-corrected band intensities using PxI system (Syngene) as described.^52^ All blots derived from the same experiment and were processed in parallel.

*RT-PCR analysis* was performed as described.^53^ Total RNA from muscle samples was extracted using the Promega SV Total RNA isolation kit. Three hundred nanogram of RNA were reverse-transcribed with SuperScript III reverse transcriptase (Life Technologies) to obtain cDNA. The cDNA was analyzed by RT-PCR (see Table S4) using SYBR Green PCR master mix (Life Technologies). Data were normalized to β2-microglobulin expression as housekeeping gene.

### Skeletal muscle proteomics

#### Protein extraction

For two-dimensional difference in gel electrophoresis (2D-DIGE) and immunoblot assays, each sample from each subject was suspended in lysis buffer (7 M urea, 2 M thiourea, 4% CHAPS, 30 mM Tris, and 1 mM PMSF) and solubilized by sonication on ice. Proteins were selectively precipitated using PlusOne 2D-Clean up Kit (GE Healthcare, Little Chalfont, UK) in order to remove nonprotein impurities, and resuspended in lysis buffer. The pH of the protein extracts was adjusted to pH 8.5 by addition of 1 M NaOH. Protein concentrations were determined by PlusOne 2D-Quant Kit (GE Healthcare).

#### 2D-DIGE

Protein minimal labeling with cyanine dyes (Cy3 and Cy5), 2D separation, and analyses were performed as described previously (for crew members A and B; SOL biopsy preflight, immediately postflight and 15 days postflight).^20^ Briefly, proteins extracted (50 µg) from each individual were labeled with Cy5, while internal standards were generated by pooling (50 µg) individual samples that were Cy3-labeled. Samples were separated on 3–10 nonlinear immobilized pH gradient (IPG) strips; the adopted gradient enables separation of protein isoforms in the first dimension, providing a detailed pattern of the muscle proteome. Each individual sample was run in triplicate (analytical replicates) to minimize intergel variability. Image analysis was performed using DeCyder 7.0 software (GE Healthcare). Statistical analysis was performed using the DeCyder 1.0 extended data analysis (EDA) module. Protein filters were set to select only those protein spots that matched > 90% of the gel images, and these protein spots were included in data analysis. Statistically significant differences of 2D-DIGE data were computed by paired one-way ANOVA (two-sided) coupled to Tukey’s multiple group comparison test; the significance level was set at *P* < 0.01. In addition, the false discovery rate (FDR) was applied as a multiple testing correction method to keep the overall error rate as low as possible.^54^ Two independent analyses were performed. Proteins of interest were identified by mass spectrometry.

#### Protein identification by mass spectrometry

Proteins were identified by peptide mass fingerprinting (PMF) utilizing a matrix-assisted laser desorption/ionization (MALDI) time-of-flight (ToF) mass spectrometer (Ultraflex III ToF/ToF; Bruker Daltonics, Bremen, Germany), as previously described.^55^ In particular, search was carried out by correlation of uninterpreted spectra to Mammalia entries in NCBInr 20090430 database (8 483 808 sequences; 2 914 572 939 residues). Where this approach was unsuccessful, additional searches were performed using electrospray ionization-MS/MS, as previously described.^56^

### Metabolomics

Blood/plasma samples were obtained both from astronauts and from a control cohort of 79 healthy volunteers. Ethylenediaminetetraacetic acid was always used as anticoagulant, but the effects of its presence were eliminated as previously described.^57^ In particular, 56 donors were collected in collaboration with the Tuscanian section of the Italian Association of Blood Donors (AVIS) in the Transfusion Service of Pistoia Hospital (Ospedale del Ceppo, AUSL 3—Pistoia, Italy), and 23 samples were collected in Bologna. Specifically, six healthy volunteers were recruited at four different times (up to 7 months, one dropped out at a specific time). No differences in the time series were found (data not shown) and all samples were merged to obtain a more heterogeneous population.

Frozen plasma samples were thawed at room temperature and shaken before use. The NMR samples were prepared according to the standard operating procedures.^58^ ^1^H-NMR spectra were acquired at 310 K using a Bruker 600 MHz spectrometer (Bruker BioSpin): water suppressed Carr–Purcell–Meiboom–Gill (CPMG) spin echo pulse sequence (RD-90°-(τ-180°-τ)n-acq) to obtain one-dimensional ^1^H-NMR spectra in which broad signals from high molecular weight metabolites (i.e. proteins and lipoproteins) are attenuated.^59^ Sixty-four FIDs were collected into 73 728 data points over a spectral width of 12 019 Hz, with a relaxation delay of 4 s and acquisition time of 3.1 s. Free induction decays were multiplied by an exponential function equivalent to a 1.0 Hz line-broadening factor before applying Fourier transformation. Transformed spectra were automatically corrected for phase and baseline distortions and calibrated (anomeric glucose doublet at 5.24 ppm) using TopSpin 3.2 (Bruker Biospin GmbH, Germany).

All metabolomic data analyses were performed using R.^60^ The spectral regions related to metabolites were assigned in the ^1^H-NMR profiles by using matching routines of AMIX 3.8.4 in combination with the BBIOREFCODE (Bruker BioSpin GmbH, Germany), and freely available datasets.^61,62^ Metabolite concentrations in arbitrary units were obtained integrating the related spectral regions. PCA was used as a first unsupervised exploratory analysis to compare metabolic profiles of astronauts and healthy volunteers, and to enable the assessment of the homogeneity or the presence of any outliers in plasma samples of volunteers that come from two different collection centers. The concentrations of each metabolite in the astronauts samples were compared with the healthy volunteers distributions using the Iglewicz and Hoaglin outlier test.^63^ *P* values < 0.01 were deemed significant and *P* values < 0.05 near-significant.

## Electronic supplementary material

Supplementary Material

## Data Availability

The datasets generated and analyzed during the current study are not publicly available due to privacy reasons but are available from the corresponding author on reasonable request.

## Electronic supplementary material

**Supplementary information** accompanies the paper on the *npj Microgravity* website (10.1038/s41526-018-0052-1).
