# Author Correction: Sarcolab pilot study into skeletal muscle’s adaptation to longterm spaceflight

**DOI:** 10.1038/s41526-018-0058-8

**Published:** 2018-10-24

**Authors:** Jörn Rittweger, Kirsten Albracht, Martin Flück, Severin Ruoss, Lorenza Brocca, Emanuela Longa, Manuela Moriggi, Olivier Seynnes, Irene Di Giulio, Leonardo Tenori, Alessia Vignoli, Miriam Capri, Cecilia Gelfi, Claudio Luchinat, Claudio Franceschi, Roberto Bottinelli, Paolo Cerretelli, Marco Narici

**Affiliations:** 1 Institute of Aerospace Medicine, German Aerospace Center (DLR), Cologne, Germany; 2 Department of Pediatrics and Adolescent Medicine, University of Cologne, Cologne, Germany; 3 Faculty of Medical Engineering and Technomathematics, FH Aachen University of Applied Science Aachen, Aachen, Germany; 4 Institute of Biomechanics and Orthopaedics, German Sport University, Cologne, Germany; 5 Department of Orthopaedics, University of Zürich, Zürich, Switzerland; 6 Department of Molecular Medicine, University of Pavia, Pavia, Italy; 7 CNR-IBFM, Segrate, MI Italy; 8 Department of Physical Performance, Norwegian School of Sport Sciences, Oslo, Norway; 9 Centre for Human and Applied Physiological Sciences, King’s College London, London, UK; 10 Department of Experimental and Clinical Medicine, University of Florence, Florence, Italy; 11 CERM Centro di Ricerca di Risonanze Magnetiche, Florence, Italy; 12 Department of Experimental, Diagnostic and Specialty Medicine, University of Bologna, Bologna, Italy; 13 Department of Biomedical Sciences for Health, University of Milan, Milano, Italy; 14 Fondazione Salvatore Maugeri (IRCSS), Scientific Institute of Pavia, Pavia, Italy; 15 Department of Biomedical Sciences, University of Padova, Padova, Italy

**Correction to**: *npj Microgravity* 10.1038/s41526-018-0052-1, Article Published online 17 Sep 2018

The original version of this Article contained an error in the spelling of the author Claudio Franceschi, which was incorrectly given as Claudio Francheschi. This has now been corrected in both the PDF and HTML versions of the Article.

