# Supplementary material for: Sarcolab pilot study into skeletal muscle’s adaptation to long-term spaceflight
Source: NPJ Microgravity. 2018 Sep 17;4:18. doi: 10.1038/s41526-018-0052-1 (PMC6141586; doi:10.1038/s41526-018-0052-1)
Supplement: Supplementary file 1 — Supplementary Material [file 41526_2018_52_MOESM1_ESM.pdf]

Supplementary Figures

Figure S1: **Regulation of vinculin isoform expression by space flight**

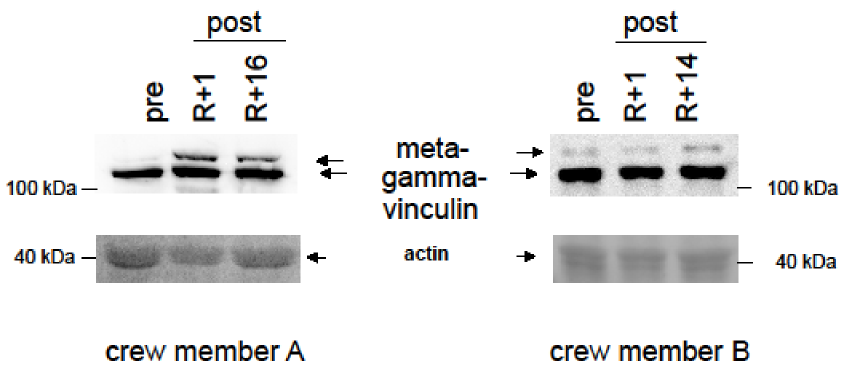

Immunoblots showing meta- and gamma-vinculin levels before and after spaceflight in both crew members. Below, loading control visualizing the staining of the skeletal alpha actin band on the membrane. kDa, kilodalton. R+1, R+14, R+16 denote 1 day, 14 and 16 days after return from space. Arrows point to the respective target protein.

Uncropped blots from Figure S1

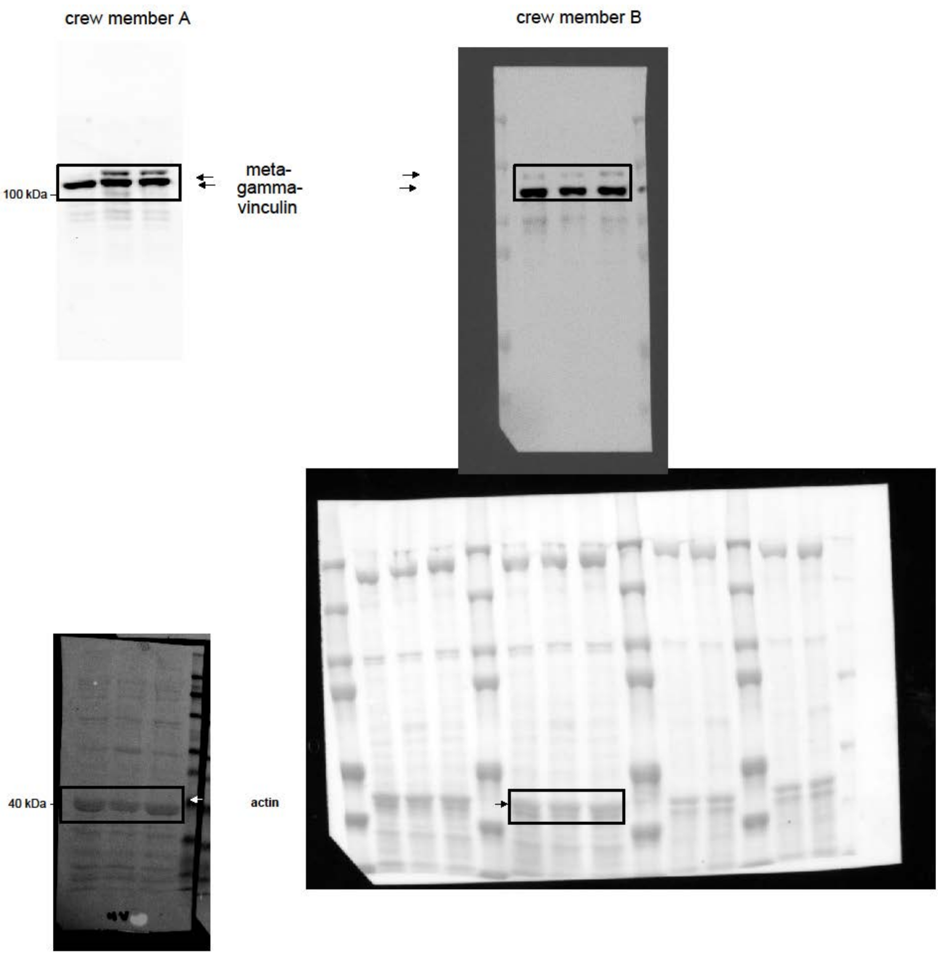

Figure S2: **Regulation of focal adhesion kinase related proteins by space flight**

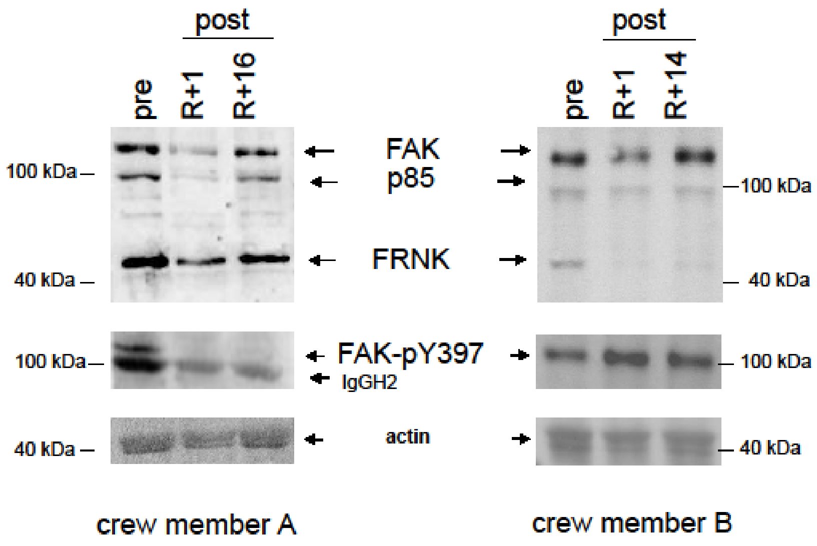

Immunoblots visualizing the expression of FAK and FRNK (top) and FAK-pY397 (middle) in the two studied crew members before and after spaceflight. Below the immunoblots, a loading control is shown, visualizing the staining of the band corresponding to skeletal alpha actin on the membrane. Abbreviations: IgGH2; heavy chain dimer of immunoglobulins. kDa, kilodalton. R+1, R+14, R+16 denote 1 day, 14 and 16 days after return from space. Arrows point to the target protein.

<u>*Uncropped blots from Figure S2*</u>

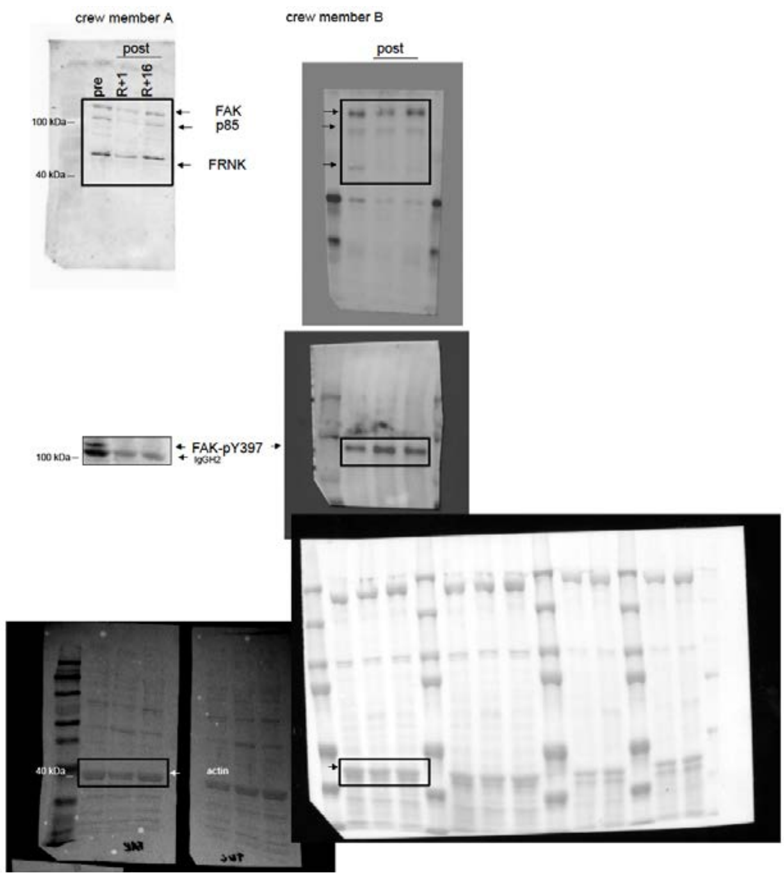

Figure S3: **Regulation of tenascin-C expression by space flight**

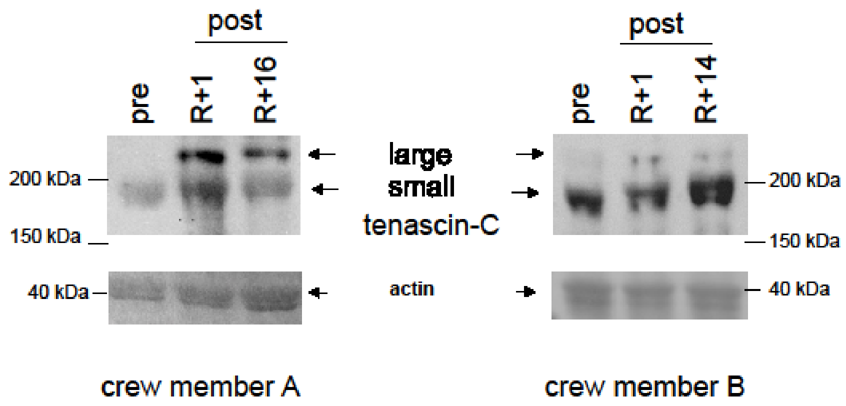

Immunoblots showing the level of the large and small tenascin-C isoform before and after spaceflight in the two crew members. Below, loading control visualizing the staining of the skeletal alpha actin band on the membrane. kDa, kilodalton. R+1, R+14, R+16 denote 1 day, 14 and 16 days after return from space. Arrows point to the respective target protein.

Uncropped blots from Figure S3

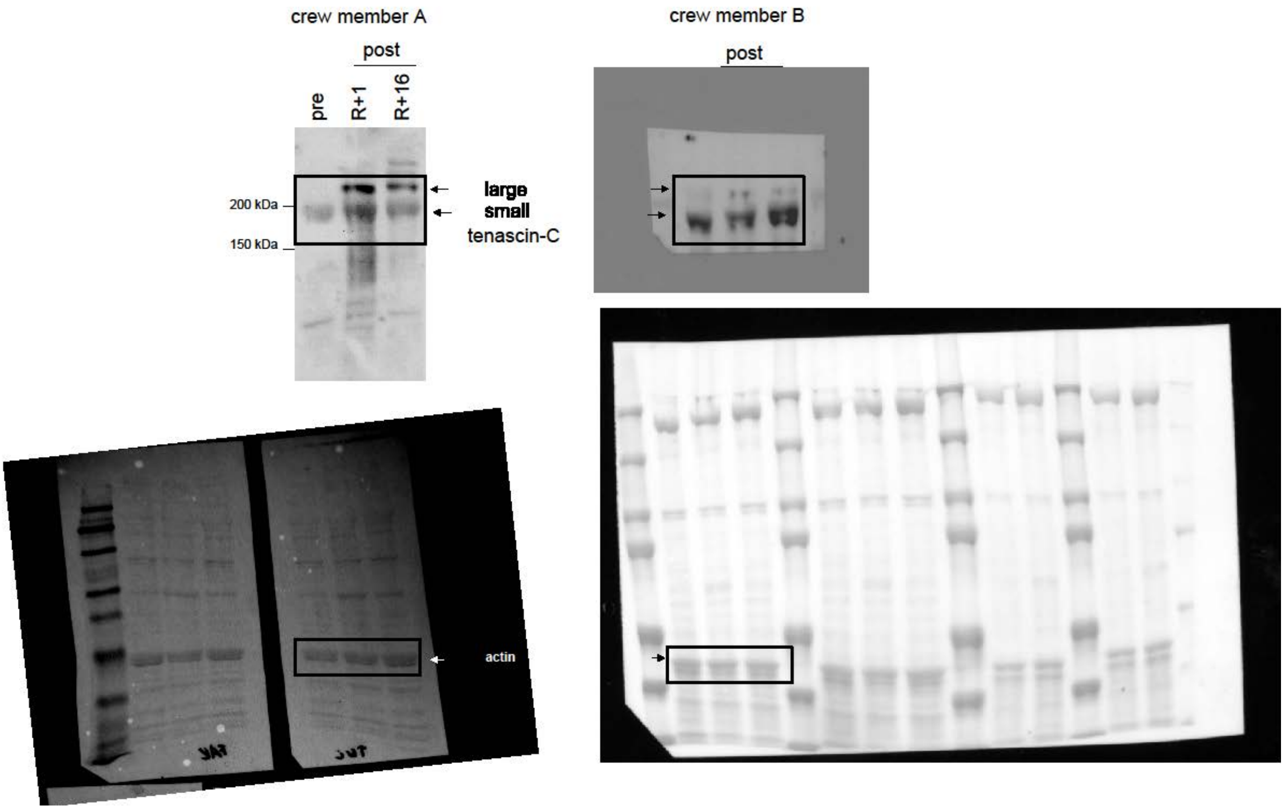

Figure S4: **Principal component analysis (PCA) of muscle proteomic data**

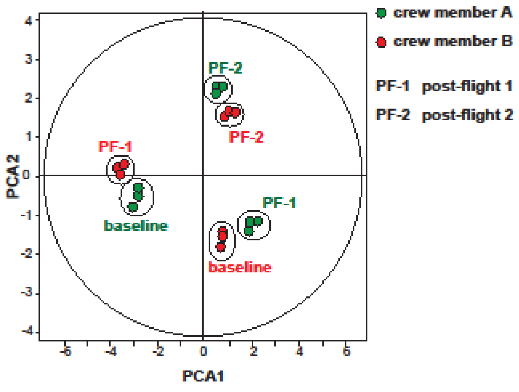

The PCA provided the overview of spots distribution in crew member A and B. In A, the protein data set of baseline was separated from PF-1 and PF-2 by PCA1 (59.2% global variation), highlighting the overall changes in protein abundance occurring immediately after flight and persisting two weeks later. PF-1 differs from PF-2 by PCA2 (25.9% global variation). Concerning B, baseline and PF-2 diverted from PF-1 through the PCA1 (61.4% global variation) suggesting their differences from PF-1. Furthermore, baseline diverted from PF-2 by PCA2 (18.5% global variation). See also Table S2.

Figure S5: **Score plot of the first three components of the PCA calculated con NMR metabolomics data.**

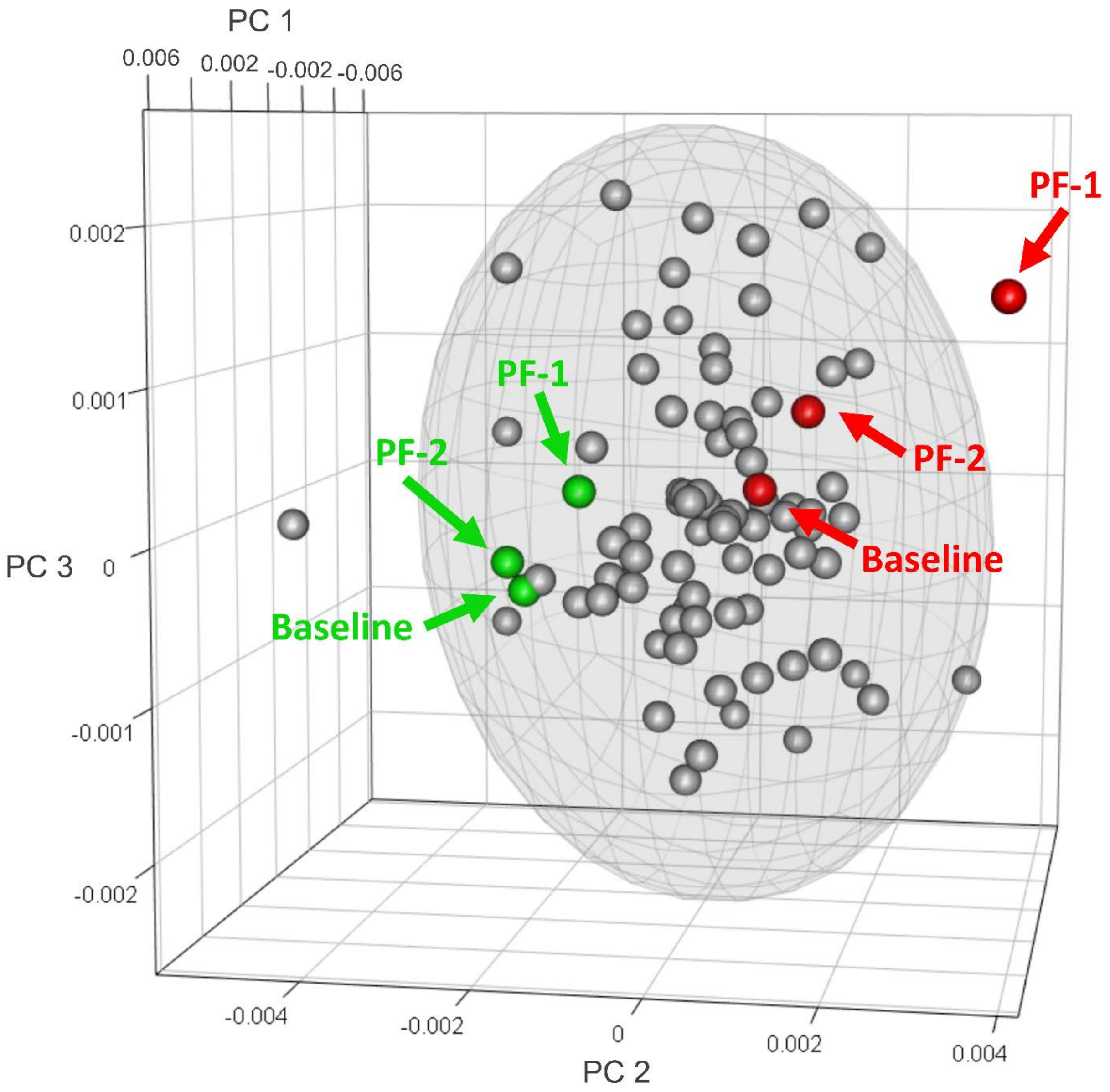

The explained variance was: PC1 55.7%, PC2 20.3%, PC3 9.6%. Each sphere represents a NMR spectrum: red crew member A, green crew member B, and grey controls. In grey the covariance ellipse.

Supplementary Tables

Table S1: **Relationships between costameric gene products, which relate to fast fiber types**

**FRNK per FAK**	***Baseline***	***PF-1***	***PF-2***
Crew member A	2.01	1.98	2.09
Crew member B	0.58	0.34	0.23
meta-vinculin per gamma-vinculin			
Crew member A	0.06	0.47	0.34
Crew member B	0.05	0.05	0.08

Note the considerable increase in meta- per gamma-vinculin content in crew member A after space flight, and the relative stability of this ratio in crew member B.

Table S2: **Proteomic analysis**

				**Crew member A**				**Crew member B**			
**Pathway**	**Protein name**	**Swiss-Prot accession number**	**Gene Symbol**	**Tukey PF-1 vs baseline**	**% fold change PF-1 vs baseline**	**Tukey PF-2 vs baseline**	**% fold change PF-2 vs baseline**	**Tukey PF-1 vs BL**	**% fold change PF-1 vs baseline**	**Tukey PF-2 vs baseline**	**% fold change PF-2 vs baseline**
**Contractile proteins**	Actin, alpha skeletal muscle	P68133	ACTA1		0	6,03E-03	-15		0		0
	Actin, alpha skeletal muscle	P68133	ACTA1		0	3,93E-03	-15	8,42E-05	19	6,72E-04	-12
	Actin, alpha skeletal muscle	P68133	ACTA1		0	2,13E-03	-12	2,29E-04	18	3,86E-03	-10
	Actin, alpha skeletal muscle	P68133	ACTA1		0	5,43E-03	-20		0	3,39E-03	-20
	Troponin I, fast skeletal muscle	P48788	TNNI2	7,44E-04	101		0		0	1,98E-03	65
	Troponin T, slow skeletal muscle (TnTs)	P13805	TNNT1	5,42E-03	-11		0	1,72E-03	19	4,10E-03	-18
	Troponin T, slow skeletal muscle (TnTs)	P13805	TNNT1	1,60E-03	-49	9,28E-03	-35	4,36E-05	42	7,04E-05	-60
	Troponin T, slow skeletal muscle (TnTs)	P13805	TNNT1		0		0	8,19E-05	32		0
	Troponin T, fast skeletal muscle (TnTf)	P45378	TNNT3	1,38E-03	124	9,35E-03	29	3,15E-04	-71		0
	Troponin T, fast skeletal muscle	P45378	TNNT3	5,21E-04	141		0	8,41E-04	-43		0
	Tropomyosin alpha-1 chain	P09493	TPM1		0		0	6,09E-05	19	1,44E-04	-17
	Tropomyosin beta chain	P07951	TPM2		0	6,18E-03	-27	3,72E-04	22		0
	Tropomyosin beta chain	P07951	TPM2		0	1,81E-03	-26	1,38E-04	18	6,52E-04	-14
**Anaerobic metabolism**	Glycogen phosphorylase, muscle form	P11217	PYGM	5,42E-03	-37		0		0		0
	Glycogen phosphorylase, muscle form	P11217	PYGM	3,23E-03	-27		0		0		0
	Glycogen phosphorylase, muscle form	P11217	PYGM	4,60E-04	-42	5,17E-03	-47		0		0
	Glycogen phosphorylase, muscle form	P11217	PYGM	7,28E-04	-47	2,26E-03	-48		0		0
	Glycerol-3-phosphate dehydrogenase [NAD(+)], cytoplasmic	P21695	GPD1	7,27E-03	21		0		0		0
	Fructose-bisphosphate aldolase A	P04075	ALDOA		0	7,09E-03	-29		0		0
	Fructose-bisphosphate aldolase A	P04075	ALDOA		0	7,45E-03	-16		0		0
	Glyceraldehyde-3-phosphate dehydrogenase	P04406	GAPDH		0		0		0	4,93E-03	15

	Glyceraldehyde-3-phosphate dehydrogenase	P04406	GAPDH		0		0		0	2,90E-03	14
	Glyceraldehyde-3-phosphate dehydrogenase	P04406	GAPDH		0		0	5,62E-04	43	1,93E-03	33
	Phosphoglycerate mutase 2	P15259	PGAM2		0		0	6,27E-03	13		0
	Alpha-enolase	P06733	ENO1		0	5,72E-03	10	2,09E-05	22		0
	Beta-enolase	P13929	ENO3		0		0	8,16E-04	21		0
	Beta-enolase	P13929	ENO3		0		0	2,56E-03	17		0
	Beta-enolase	P13929	ENO3	1,47E-03	17	1,32E-03	19	2,86E-03	13	6,39E-03	11
	Pyruvate kinase PKM	P14618	PKM		0		0	2,53E-03	19		0
	L-lactate dehydrogenase A chain	P00338	LDHA		0	7,53E-03	-29		0		0
**Aerobic metabolism**	Aconitate hydratase, mitochondrial	Q99798	ACO2	4,32E-03	-25	7,27E-03	-24		0		0
	Isocitrate dehydrogenase [NADP], mitochondrial (IDH)	P48735	IDH2		0		0	1,59E-03	-17	4,57E-03	-13
	Isocitrate dehydrogenase [NADP], mitochondrial (IDH)	P48735	IDH2		0		0	2,66E-04	-33	1,68E-03	-19
	2-oxoglutarate dehydrogenase, mitochondrial	Q02218	OGDH	4,25E-03	-26		0		0		0
	2-oxoglutarate dehydrogenase, mitochondrial	Q02218	OGDH	5,31E-03	-22		0		0		0
	Dihydrolipoyl dehydrogenase, mitochondrial	P09622	DLD	6,31E-03	-22	4,40E-03	-21	9,07E-03	-40		0
	Succinyl-CoA ligase [ADP-forming] subunit beta, mitochondrial	Q9P2R7	SUCLA2		0		0	2,85E-03	-26		0
	Succinate dehydrogenase [ubiquinone] flavoprotein subunit, mitochondrial	P31040	SDHA		0	3,75E-03	-33	1,21E-03	-46	7,36E-03	-26
	Malate dehydrogenase, cytoplasmic	P40925	MDH1	2,03E-04	-27	2,28E-03	-24	1,10E-03	-18	1,70E-03	-16
	Malate dehydrogenase, cytoplasmic	P40925	MDH1	2,91E-03	-32	2,13E-03	-39		0		0
	Aspartate aminotransferase, cytoplasmic	P17174	GOT1	7,43E-03	-22	2,13E-03	-34	5,82E-04	-22	9,14E-04	-20
	Aspartate aminotransferase, cytoplasmic	P17174	GOT1	3,08E-03	-17	1,81E-03	-25	5,24E-03	-27	5,91E-03	-26
	Aspartate aminotransferase, cytoplasmic	P17174	GOT1		0	4,41E-03	-42		0		0
	Aspartate aminotransferase, mitochondrial (mAspAT)	P00505	GOT2		0		0	6,73E-03	-37		0
	NADH dehydrogenase [ubiquinone] iron-sulfur protein 3, mitochondrial	O75489	NDUFS3	1,57E-03	41	3,78E-03	58	1,12E-04	-32	4,96E-03	-11

	NADH dehydrogenase [ubiquinone] flavoprotein 1, mitochondrial	P49821	NDUFV1		0		0	2,35E-03	18		0
	Cytochrome b-c1 complex subunit 1, mitochondrial	P31930	UQCRC1	1,38E-03	-19	8,23E-04	-28	6,13E-04	-32		0
	ATP synthase subunit alpha, mitochondrial	P25705	ATP5A1	1,16E-03	-21	7,13E-04	-19		0		0
	ATP synthase subunit beta, mitochondrial	P06576	ATP5B	1,21E-03	-31	3,80E-03	-31		0		0
	ATP synthase subunit beta, mitochondrial	P06576	ATP5B	1,58E-03	-26	3,82E-03	-24	3,57E-04	-25	3,32E-03	-13
	ATP synthase subunit d, mitochondrial	O75947	ATP5H	5,99E-03	-12	3,23E-04	-20	1,53E-03	-43		0
	Electron transfer flavoprotein subunit beta	P38117	ETFB		0		0	3,22E-03	-39		0
	Very long-chain specific acyl-CoA dehydrogenase, mitochondrial	P49748	ACADVL	7,03E-03	-24		0	3,40E-03	-46		0
	Short-chain specific acyl-CoA dehydrogenase, mitochondrial	P16219	ACADS	3,60E-03	-32	5,14E-03	-30	1,21E-04	-81	6,63E-03	-23
	Enoyl-CoA delta isomerase 1, mitochondrial	P42126	ECI1		0	8,02E-03	-15		0		0
	Delta(3,5)-Delta(2,4)-dienoyl-CoA isomerase, mitochondrial	Q13011	ECH1		0		0	4,91E-05	-72		0
**energy transduction**	Creatine kinase M-type	P06732	CKM	3,13E-03	-19	6,54E-03	-28		0		0
	Creatine kinase M-type	P06732	CKM	4,60E-04	-21	3,78E-03	-28	2,24E-03	-10	6,12E-04	-14
	Creatine kinase M-type	P06732	CKM	2,54E-03	-26	5,83E-03	-26	1,33E-04	-11	3,67E-05	-16
	Creatine kinase M-type	P06732	CKM	4,64E-03	-23	7,09E-03	-28	2,45E-03	-12	9,17E-04	-16
	Creatine kinase S-type, mitochondrial	P17540	CKMT2	4,64E-03	-29	4,56E-03	-32	3,60E-03	-28		0
	Adenylate kinase isoenzyme 1	P00568	AK1		0	3,15E-03	38		0		0

The table lists those spots that changed from Baseline to PF-1 and PF-2.

Table S3: **Testing schedule**

**Crew Member**	**Test**	**Baseline**	**Post-Flight 1 (PF-1)**	**Post-Flight 2 (PF-2)**
A	Blood Sample & Biopsy	L-79	R+0	R+15
	Magnetic Resonance Imaging	L-79	R+3	R+15
	Plantar flexor testing	L-79	R+1	R+15
B	Blood Sample & Biopsy	L-76	R+0	R+15
	Magnetic Resonance Imaging	L-76	R+3	R+15
	Plantar flexor testing	L-76	R+4	R+15

Baseline dates are relative to launch (L), and post-flight dates are relative to return to Earth (R+0 = landing day).

Table S4: **Forward and reverse primers used for RT-PCR**

Gene	Forward primer	Reverse primer
MuRF-1	5'–CCTGAGAGCCATTGACTTTGG–3'	5'–CTTCCCTTCTGTGGACTCTTCCT–3'
Atrogin-1	5'-GCAGCTGAACAACATTCAGATCAC–3'	5'-CAGCCTCTGCATGATGTTCAGT-3'
p62	5'–GCTTCCAGGCGCACTACC–3'	5'–CATCCTCACGTAGGACATGG–3'
Beclin-1	5'–TGGAAGGGTCTAAGACGT–3'	5'–GGCTGTGGTAAGTAATGGA–3'
PGC1alpha	5'-CAGGATTTCATCTGAGTGTGGA-3'	5'-GCGAGAGAGAAAGGAAAAGAACAA-3'
SREBP-1	5'–CGTCCCTCCTGTTGTGAAAT	5'–AGTGTTTTCACGGGACCAAG–3'
NRF2	5'-CACAGAAGACCCCAACCAGT-3'	5'-CTGTGCTTTCAGGGTGGTTT- 3'
β2-microglobulin	5'-GCTGTGCTCGCGCTACTCTCTCT-3'	5'-TCTGCTGGATGACGTGAGTAAAC- 3'
